# The Utility and Acceptability of a New Noninvasive Ventilatory Assist Device, Rest-Activity Cycler-Positive Airways Pressure, During Exercise in a Population of Healthy Adults: Cohort Study

**DOI:** 10.2196/35494

**Published:** 2022-08-01

**Authors:** Julie Reeve, Sarah Mooney, Nicola Jepsen, David White

**Affiliations:** 1 School of Clinical Sciences Auckland University of Technology Auckland New Zealand; 2 Department of Mechanical Engineering Auckland University of Technology Auckland New Zealand

**Keywords:** noninvasive ventilation, exercise, feasibility, humans, COPD, physiotherapy, pulmonary rehabilitation, rehabilitation

## Abstract

**Background:**

Noninvasive ventilation has been demonstrated to benefit people who have moderate to severe chronic obstructive pulmonary disease during acute exacerbations. Studies have begun to investigate the effectiveness of noninvasive ventilation during pulmonary rehabilitation to improve outcomes for people with chronic obstructive pulmonary disease; however, the lack of portability and humidification of these devices means their use is limited, especially when performing activities of daily living. A new prototype device, RACer-PAP (rest-activity cycler-positive airways pressure), delivers battery-operated positive airway pressure via a nasal interface while regulating nasal airway apportionment bias, removing the need for supplementary humidification. This device may offer people with chronic obstructive pulmonary disease an improved ability to participate in pulmonary rehabilitation and activities of daily living.

**Objective:**

To assess the feasibility of exercising with the RACer-PAP in situ and the acceptability of the device during exercise in normal, healthy individuals.

**Methods:**

A total of 15 healthy adults were invited to attend 2 exercise sessions, each 1 week apart. Sessions lasted approximately 1 hour and included 2 baseline 6-minute walk distance assessments, once with and once without the RACer-PAP in situ. Vital signs and spirometry results were monitored throughout, and spirometry was performed pre- and posttesting with RACer-PAP. Subjective questionnaires ascertained participant feedback on exercising with the device in situ.

**Results:**

Of the 15 initial participants, 14 (93%) completed both sessions. There were no adverse events associated with exercising with the device in situ. There were no differences in vital signs or 6-minute walk distance whether exercising with or without the device in situ. There were small increases in maximum dyspnea score (on the Borg scale) when exercising with the device in situ (median score 2.0, IQR 0.5-3.0, vs 3.0, IQR 2.0-3.25). There were small increases in forced vital capacity following exercise with the RACer-PAP. None of the participants reported symptoms associated with airway drying. Participant feedback provided recommendations for modifications for the next iteration of the device prior to piloting the device with people with chronic obstructive pulmonary disease.

**Conclusions:**

This study has shown RACer-PAP to be safe and feasible to use during exercise and has provided feedback for modifications to the device to improve its use during exercise. We now propose to consider the application of the device in a small pilot feasibility study to assess the safety, feasibility, and utility of the device in a population of people with moderate to severe chronic obstructive pulmonary disease.

**Trial Registration:**

Australian New Zealand Clinical Trials Registry ACTRN12619000478112; https://www.anzctr.org.au/Trial/Registration/TrialReview.aspx?id=375477

## Introduction

Chronic obstructive pulmonary disease (COPD) is a term for progressive chronic lung diseases that cause airflow limitation, including emphysema, chronic bronchitis, and chronic asthma [1]. Based on large epidemiological studies, the global prevalence of COPD is estimated to be around 11.7% (95% CI 8.4%-15%), with around 3 million deaths occurring annually [2]. It is the fourth leading cause of mortality worldwide [1]. Guidelines for the management of COPD support a combination of interventions, including pharmacological therapy, smoking cessation, self-management of exacerbations, and pulmonary rehabilitation (PR) to improve health outcomes. Pulmonary rehabilitation is widely considered the gold standard intervention in patients with COPD to reduce dyspnea, improve exercise capacity, and improve health-related quality of life (QOL) [3].

Noninvasive ventilation (NIV) has been demonstrated to benefit people who have moderate to severe COPD during acute exacerbations and can help to reduce respiratory rate, mortality and intubation rates, and improve arterial oxygenation [4,5]. Studies have begun to investigate the effectiveness of NIV during exercise or PR to improve outcomes for patients with COPD. A recent meta-analysis [4] investigating NIV during exercise training found that NIV may help people with COPD to exercise at a greater intensity and duration and to achieve better training results compared to exercise training alone or exercise with sham NIV.

Most NIV devices are impractical for undertaking everyday activities and exercise because they are large, heavy, expensive, and rely on an AC power source. A further problem with many devices is the lack of humidification of inspired gases, which can dry the airways and airway secretions and cause problems for people with COPD. Additionally, most devices use a face mask interface, which is often unacceptably claustrophobic during exercise for people with COPD [6]. A lightweight, portable, humidified NIV device with a more user-friendly interface has the potential to improve PR outcomes and impact the lives of those with COPD who are restricted in their day-to-day lives due to their reduced exercise tolerance and breathlessness.

The RACer-PAP (rest activity cycler-positive airway pressure) is an NIV device designed by author DW and his design team at the BioDesign Laboratory of the Auckland University of Technology, which specializes in biomedical engineering. The prototype device was originally designed to increase comfort for patients with sleep apnea. It has been safely tested in a small sample of this population and has been found to reduce drying of the airways and nasal congestion [7]. The prototype RACer-PAP operates on room air and works in a similar manner to a continuous positive airway pressure (CPAP) machine. CPAP is a widely utilized form of NIV and has been shown to splint open the airways at end expiration, counter intrinsic positive end-expiratory pressure (PEEP), and reduce the work of breathing [6,8]. Use of CPAP during exercise has also been shown to reduce breathlessness and improve exercise tolerance in people with COPD [6,9,10]. However, while using CPAP, the nasal cycle (where one nostril periodically conducts a greater apportionment of tidal airflow than the other [11]) is abolished [12], leading to airway drying [13,14]. Current CPAP machines use supplementary humidification to prevent this airway drying, which is impractical if using the device during travel or mobility. The prototype RACer-PAP device delivers the same positive airway pressure to the nose as a CPAP machine while simultaneously regulating nasal airflow apportionment bias. This device effectively reinstates the body’s natural air-conditioning and protection systems and removes the need for supplementary humidification [15]. RACer-PAP technology, if acceptable to people with COPD, may be useful for applying positive airway pressure during travel, exercise, and activities of daily living.

**Figure 1:**
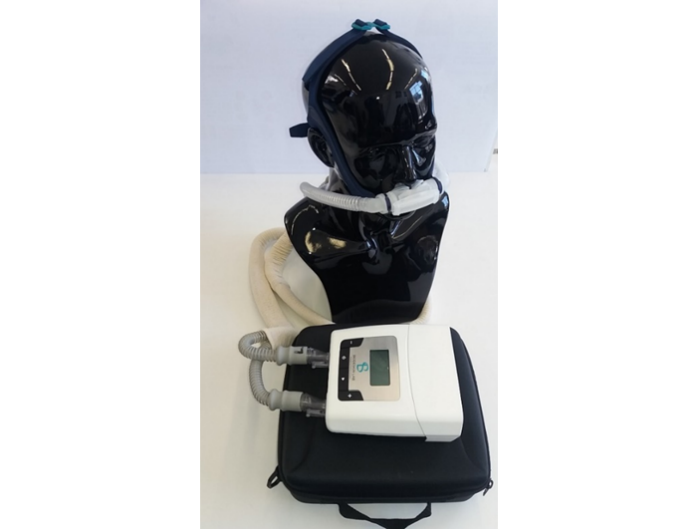
RACer-PAP prototype in situ.

The prototype RACer-PAP uses nasal pillows (Figure 1) as the interface. While the nasal breathing cycle is not fully understood, it is thought that, under usual circumstances, one nostril allows more airflow to pass through than the other, with flow alternating between nostrils approximately every three hours [11]. This is caused by periodic unilateral obstruction by turbinate hypertrophy and is believed to aid in the removal of contaminants [11]. A unique feature of the RACer-PAP is that the device determines the natural flow-dominant nostril for each individual within its first few assisted breaths, and following this, the device ramps up to an operator-set positive pressure to ensure that the dominant nostril passes a higher airflow than the nondominant nostril. PEEP is adjusted to each person’s comfort level, with a range from 6 to 20 cm H_2_O, and accommodates each person’s intrinsic PEEP [8]. This airflow bias between nostrils continues for a preset time, then switches, so that the other nostril receives the higher flow rate. This cycle time is predetermined by the therapist. The device can deliver up to 73 liters per minute of room air through each of the hoses (via each side of the nasal pillow), ensuring that the device is able to meet the high air flow demands of users, even during exercise. Through this process, the RACer-PAP device eliminates the need for supplementary humidification. The device can be battery operated, offering the convenience of treatment portability. We believe the RACer-PAP may have the potential to improve the ability of people with moderate to severe COPD to participate in PR and in activities of daily living. Prior to testing the acceptability, utility, and effectiveness of this device in people with COPD, the prototype RACer-PAP device requires evaluation in healthy volunteers.

The aims of this study were to (1) assess the feasibility of exercising with the RACer-PAP prototype in situ; (2) investigate the utility and acceptability of the RACer-PAP prototype during rest and exercise; and (3) identify potential safety issues while utilizing the RACer-PAP prototype during exercise.

## Methods

### Ethics Approval

Ethical permission for the study was granted by the Health and Disability Ethics Committee of New Zealand on December 5, 2018 (study number 18/NTB/191). Institutional ethics approval was granted by the Auckland University of Technology Ethics Committee on April 15, 2019 (study number 19/129). The study was prospectively registered and approved on ANZCTR (ACTRN12619000478112) on March 22, 2019.

### Study Design

This was a feasibility study to establish the utility and acceptability of the prototype RACer-PAP device during exercise in normal, healthy individuals. Participants individually attended 2 sessions at the Auckland University of Technology (Auckland, NZ) that were held a maximum of 1 week apart. At session 1, participants completed baseline screening and became familiar with the prototype RACer-PAP device and the 6-minute walk test (6MWT). At session 2, participants completed exercise testing with and without the prototype RACer-PAP in situ in randomized order and provided feedback on exercise with the RACer-PAP. Both sessions were at a similar time of day (to negate circadian variability) and lasted a maximum of 1.5 hours.

### Participants

Participants were purposefully selected to include a diversity of ages, sexes, and ethnicities. Subjects were included if they were healthy adults aged >25 years and were able to attend both scheduled sessions. Subjects were excluded if they had facial deformities, nasal polyps, or turbinate abnormalities, such as a sinus infection or other conditions, that might have influenced nasal airflow regulation; were unwilling to wear the device or unable to tolerate the nasal pillow interface; had a diagnosis of heart disease, high blood pressure, respiratory disease, or any illness or injury that impaired physical performance; had an active infection; had positive findings from the Physical Activity Readiness Questionnaire (PAR-Q) and Electronic Physical Activity Readiness Medical Examination (ePARmed-X+) risk assessment tools; were under advice from a medical practitioner to avoid exercise; had spirometry results indicating airflow obstruction, with a forced expiratory volume in 1 second (FEV1)/forced vital capacity (FVC) ratio of less than 70% [1]; or were unable to understand written or spoken English (this study lacked funding for translators).

### Procedures

Two health care professionals were present at each session. The participants were screened for their suitability to participate using the PAR-Q risk assessment tool. If the PAR-Q result was positive, the participant completed an ePARmed-X+ [16] assessment to determine if they required referral to a medical professional for exercise clearance. If a participant was eligible to take part in the study, baseline screening of vital signs (heart rate, blood pressure, oxygen saturation) and spirometry (FEV1, FVC, and FEV1/FVC ratio) were undertaken. A trial 6MWT was undertaken following best practice guidelines [17]. This was followed by a 30-minute rest and was then followed by a second 6MWT.

**Figure 2:**
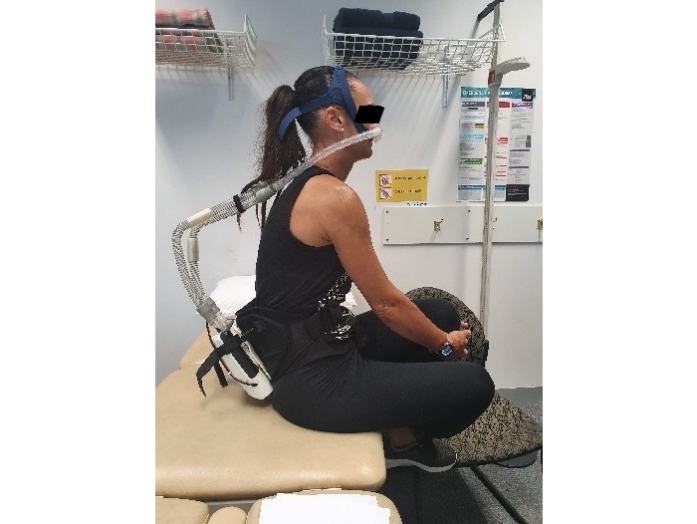
RACer-PAP at rest (side view).

**Figure 3:**
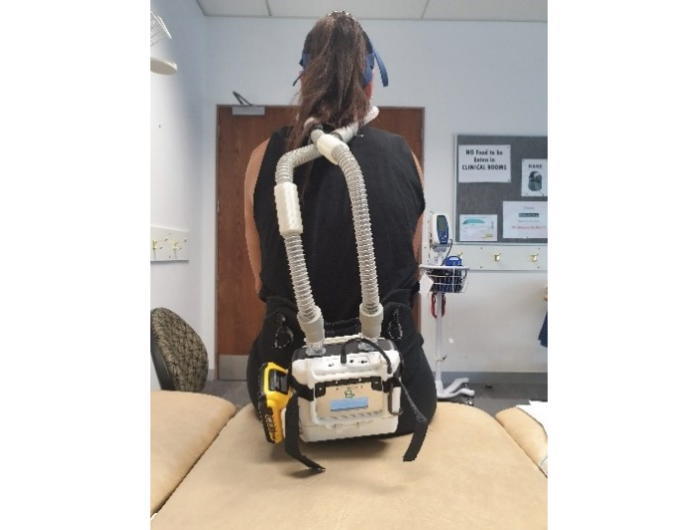
RACer-PAP at rest (posterior view).

**Figure 4:**
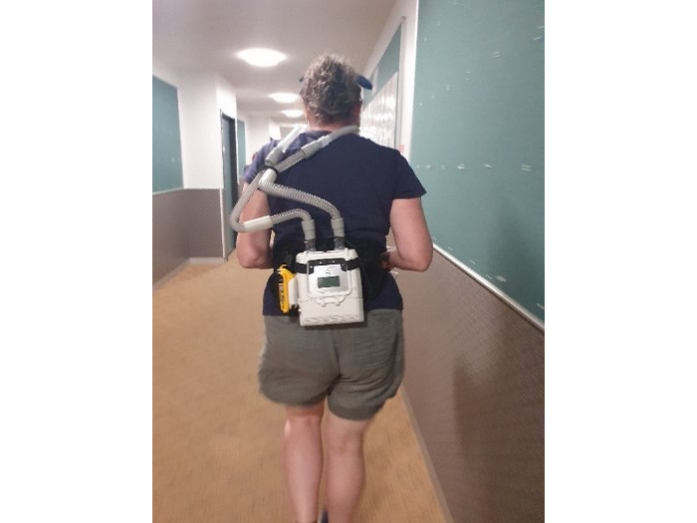
RACer-PAP during exercise.

Following baseline testing, the participant was shown the RACer-PAP, the device was explained, and the participant was fitted with the device at rest and during exercise (see Figure 2, Figure 3, and Figure 4). The device was worn at rest for 10 minutes at a participant-selected PEEP level between 6 to 10 cm H_2_O. The participant-selected PEEP level was noted for further testing purposes at session 2. Participants were instructed to nose breathe, if possible, but to mouth breathe when necessary.

Immediately following removal of the RACer-PAP, spirometry testing and vital sign measurement were undertaken and the “RACer-PAP at rest” questionnaire was completed. Participants were allocated their own RACer-PAP nasal interface and tubing, which were sterilized and used for both assessments. After 1 week, the participants underwent baseline testing of vital signs and spirometry (as per session 1) and then completed two 6MWT assessments, one with the PACer-PAP in situ, and one without. The order in which these assessments were undertaken was randomized using computer-generated numbers to wash out any order effect. The allocation of the first assessment was stored in a sealed envelope and was either 6MWT with RACer-PAP in situ at the participant-determined comfortable PEEP level or 6MWT without RACer-PAP in situ. Immediately following the 6MWT, vital signs and spirometry were assessed. Participants then had a 30-minute rest, completed the second 6MWT, and underwent spirometry and vital sign measurements. The second RACer-PAP questionnaire (on exercise) was completed prior to the end of the session, when participants were encouraged to provide feedback through a Likert scale and an open-ended question requesting “any other comments.”

### Data Analysis

As this was a small feasibility study (N=15), the only reason to conduct statistical testing was to ascertain a measure of variance and within-subject differences. Demographic data were analyzed using descriptive statistics. Normally distributed data were described using the mean (SD) and nonnormally distributed data using the median (IQR). Data were analyzed for within-subject differences using paired-sample 2-tailed *t* tests to determine any differences in interval or ratio measures with and without the RACer-PAP in situ. The Wilcoxon signed-rank test was used to analyze nonparametric data. Results of the Likert scale questions about the acceptability and comfort of the device were collated, and open comments were themed for commonality.

## Results

### Participants

Fifteen participants were recruited via display posters at the Auckland University of Technology between December 2019 and December 2020. Fifteen participants attended session 1. One participant dropped out following session 1 (no reason for the dropout was given); thus, session 2 was attended by 14 participants. Although there was an 18-week study shutdown period in the middle of data collection due to COVID-19 lockdowns, the target sample size was achieved. The authors consider that the data from the sample of 15 participants is adequate to provide useful information about the feasibility, usability, and acceptability of this device [18]. Baseline characteristics of the participants are shown in Table 1.

**Table 1:** Baseline characteristics of all participants (N=15).

Characteristics		Values
**Sex, n (%)**		
	Female	6 (40)
	Male	9 (60)
**Ethnicity, n (%)**		
	New Zealander European	8 (54)
	Māori	2 (13)
	Asian	2 (13)
	Pacific Peoples	1 (7)
	European	1 (7)
	Other	1 (7)
Age (years), mean (SD, range)		50.6 (12.6, 26-68)
Height (cm), mean (SD, range)		171.4 (9.2, 154-184)
Weight (kg), mean (SD, range)		79.7 (15.7, 51.9-105.8)
Resting heart rate (bpm), mean (SD, range)		74.2 (12.9, 55-100)
Systolic blood pressure (mm Hg), mean (SD, range)		127.9 (14.9, 105-152)
Diastolic blood pressure (mm Hg), mean (SD, range)		79.1 (8.4, 66-99)
Resting SpO_2_ (%), mean (SD, range)		97.4 (1.5, 95-100)
Forced expiratory volume in 1 second (L/min), mean (SD, range)		3.14 (0.77, 1.57-4.76)
Forced vital capacity (L/min), mean (SD, range)		3.95 (0.98, 2.28-5.87)
Forced expiratory volume in 1 second/forced vital capacity ratio, mean (SD, range)		0.79 (0.06, 0.69-0.92)
6-minute walk distance (meters), mean (SD, range)		651.3 (86.6, 490-751)

### Outcomes

Table 2 shows outcomes following 6MWT with and without the RACer-PAP in situ at session 2. The Wilcoxon signed-rank test revealed a significant increase in maximum dyspnea experienced during the 6MWT with the RACer-PAP in situ, with a moderate effect size (*r*=-0.45). A significant increase in FVC was seen following the 6MWT with the RACer-PAP in situ (Table 2). There were no significant changes in any other outcomes measured during this testing.

**Table 2:** Outcomes following 6-minute walk test with and without the device (rest-activity cycler-positive airways pressure) in situ.

Outcome	Subjects, n	After test 1 (without RACer-PAP^a^)	After test 2 (with RACer-PAP)	Test statistic	*P* value
Six-minute walk distance (meters), mean (SD)	14	657.9 (109.5)	653.5 (103.4)	t_13_=0.274	.79
Forced expiratory volume in 1 second (L/min), mean (SD)	12^b^	3.19 (0.68)	3.18 (0.79)	t_11_=0.237	.82
Forced vital capacity (L/min), mean (SD)	12^b^	3.95 (0.98)	4.11 (1.02)	t_11_=–2.506	.03
Forced expiratory volume in 1 second/forced vital capacity ratio, mean (SD)	12^b^	0.81 (0.11)	0.77 (0.03)	t_11_=1.294	.22
Resting heart rate (bpm), mean (SD)	14	87.1 (18.6)	91.3 (16.3)	t_13_=–1.537	.15
Systolic blood pressure (mm Hg), mean (SD)	8^b^	129 (9.8)	132 (11.1)	*t*_7_=–1.240	.26
Diastolic blood pressure (mm Hg), mean (SD)	8^b^	81.9 (5.1)	84.1 (6.3)	*t*_6_=–0.949	.38
Resting SpO_2_ (%), mean (SD)	14	97.8 (0.89)	97.6 (1.3)	t_13_=0.715	.49
Maximum dyspnea (Borg scale), median (IQR)	14	2.0 (0.5-3.0)	3.0 (2.0-3.25)	Z=–2.41	.02

^a^RACer-PAP: rest-activity cycler-positive airways pressure

^b^The number of participants was lower for these outcomes, as data were unavailable due to equipment error, malfunction, or poor participant technique.

There were no adverse events at any time during the testing period and no participants asked for the RACer-PAP to be removed at any point. Participants were asked to select their own breathing pressure (range 6-10 cm H_2_O). Six of 15 participants (40%) selected 6 cm H_2_O pressure, 3/15 participants (20%) selected 7 cm H_2_O, 4/15 participants (27%) selected 8 cm H_2_O, and 2/15 participants (13%) selected 10 cm H_2_O. The mean pressure selected was 7.3 (SD 1.4) cm H_2_O.

Participants were asked to rate the utility and comfort of the RACer-PAP at rest and during exercise using a Likert scale. The results are shown in Table 3 and Table 4.

**Table 3:** Likert scale ranking of utility and comfort of the device (rest-activity cycler-positive airways pressure) at rest (N=15).

Question	Scale	Participant ratings, n (%)						Rank mode (mean)
		1	2	3	4	5		
How easy was it to fit the device?	Very easy (1) to very difficult (5)	0	5 (33)	5 (33)	5 (33)	0	2 (2.93)	
How do you find wearing the device?	Very comfortable (1) to very uncomfortable (5)	0	8 (53)	4 (27)	2 (13)	1 (7)	2 (2.63)	
How would you rate the overall comfort of wearing this device?	Very comfortable (1) to very uncomfortable (5)	0	7 (47)	5 (33)	3 (20)	0	2 (2.7)	
How well does the device fit at rest?	Very well (1) to not well at all (5)	1 (7)	9 (60)	3 (20)	2 (13)	0	2 (2.4)	
How would you rate the comfort of the waist strap?	Very comfortable (1) to very uncomfortable (5)	3 (20)	7 (47)	1 (7)	2 (13)	1 (7)	2 (2.29)	
How would you rate the overall comfort with the nasal mask whilst wearing this device?	Very comfortable (1) to very uncomfortable (5)	0	6 (40)	5 (33)	4 (27)	0	2 (2.77)	
How do you rate the weight of the device?	Very light (1) to very heavy (5)	2 (13)	2 (13)	5 (33)	6 (40)	0	3 (2.93)	
How would you rate your overall ability to breathe whilst wearing the device at rest?	Very easy (1) to very difficult (5)	1 (7)	4 (27)	5 (33)	5 (33)	0	3 (2.90)	
How would you rate the dryness in your nose (mouth) whilst wearing the device at rest?	Very moist (1) to very dry (5)	1 (7)	3 (20)	9 (60)	1 (7)	1 (7)	3 (2.83)	

**Table 4:** Likert scale ranking of utility and comfort of the device (rest-activity cycler-positive airways pressure) during exercise (N=14).

Question	Scale	Participant ratings, n (%)					Rank mode (mean)
		1	2	3	4	5	
How did you find the weight of the device during exercise?	Very light (1) to very heavy (5)	0	4 (29)	5 (36)	4 (29)	1 (7)	3 (3.10)
How did you rate the overall portability of the device during exercise?	Very portable (1) to not portable (5)	4 (29)	3 (22)	1 (7)	5 (36)	1 (7)	4 (2.71)
How did you rate the overall comfort of the nasal mask during exercise?	Very comfortable (1) to very uncomfortable (5)	1 (7)	5 (36)	3 (22)	5 (36)	0	2 and 4 (2.82)
How did you rate the overall comfort of the waist strap during exercise?	Very comfortable (1) to very uncomfortable (5)	1 (7)	4 (29)	7 (50)	1 (7)	1 (7)	3 (2.75)
How do you rate your overall ability to move whilst exercising with the device compared to exercising without the device?	Much easier (1) to much harder (5)	0	0	3 (21)	11 (79)	0	4 (3.71)
How stable did the device feel during exercise?	Very stable (1) to very unstable (5)	0	7 (50)	5 (36)	1 (7)	1 (7)	2 (2.64)
How do you rate your overall ability to breathe while exercising with the device, compared to exercising without the device?	Much easier (1) to much harder (5)	0	0	0	11 (79)	3 (21)	4 (4.14)
How do you rate your overall ability to exercise with the device, compared to exercising without the device?	Much easier (1) to much harder (5)	0	2 (14)	2 (14)	8 (57)	2 (14)	4 (3.68)
How would you rate the dryness in your nose (mouth whilst wearing the device during exercise?	Very moist (1) to very dry (5)	0	7 (50)	3 (21)	2 (14)	2 (14)	2 (2.93)

### Open Comments

Participant feedback was obtained both at rest (during session 1) and following exercise (during session 2). At both time points, comments centered around 3 emergent themes: the device and related interfaces, the effect of the device on breathing, and recommendations for future use.

#### Session 1: Device and Related Interfaces (RACer-PAP at Rest)

The weight of the device was a dominant theme, with several participants describing the weight of the device as unfavorable. A smaller device was recommended to enhance the clinical utility of the device and allow future users to use the device more discreetly. Participants also suggested that shorter, less bulky tubing would be desirable. The nasal interface was described by a small number of participants as uncomfortable, causing their noses to become wet.

#### Session 1: Device Effect on Breathing (RACer-PAP at Rest)

Several participants commented on the effect of the device on their breathing. One participant reported that their breathing was easier, one reported that their breathing felt “strange,” resulting in increased awareness, one reported difficulty synchronizing their breathing at rest, and one person described the removal of the device as resulting in “...a wave of relaxed sensation lasting 5 seconds.”

#### Session 2: Device and Related Interface (RACer-PAP After Exercise)

The weight of the device was again considered too heavy and potentially cumbersome, with some participants recommending a smaller device. Participants found that the device bounced against the lower back, noting that improved stabilization of the device was necessary. Some participants felt that the belt with the device in situ felt “unbalanced,” requiring frequent adjustment. Some suggested that this might impact breathing or cause discomfort during exercise. Some comments noted that the tubing was too long and that the nasal interface was uncomfortable. A softer, smaller, more discreet interface was suggested for use during activities of daily living. Some participants also noted mild discomfort during exercise in relation to the air temperature: they experienced nostril dampness and their spectacles steamed up.

#### Session 2: Device Effect on Breathing (RACer-PAP After Exercise)

Several participants commented on the effect of the device on their breathing during exercise. For some, breathing required increased awareness and effort, especially during the expiratory phase. One participant described difficulty with nose breathing during exercise.

### Recommendations for Further Use

Suggestions from participants included reduced device operating noise and a smaller, lighter device, which would be more discreet and aesthetically pleasing when undertaking activities. They also suggested that improved portability and flexibility of the interfaces (tubing, head strap, and nasal interface) would improve the usability of the device. It was also recommended that the device be simple and compact, to ensure that individuals can assemble and put on the device independently.

## Discussion

This small study found that in healthy individuals, exercising with the RACer-PAP in situ was safe, feasible, and acceptable to participants. Suggestions to increase comfort and utility of the device for exercise rehabilitation purposes and everyday activity were provided and will enable the development team to make ongoing modifications to the device.

Enabling people with respiratory disease to improve exercise capabilities, reduce dyspnea, and improve QOL has been the focus of PR for several decades. High quality evidence has shown PR to be a cornerstone intervention in achieving such outcomes [1], but patients with severe to very severe COPD may have difficulty achieving a sufficient training intensity with PR to achieve improvements in outcomes [19]. In a Cochrane review undertaken in 2014 [4], the use of NIV during PR was found to be safe; it improved exercise tolerance and dyspnea in a single treatment session, but evidence of improvement compared to controls was less consistent with longer-term training. It is currently unclear whether the demonstrated benefits of NIV during exercise training are clinically worthwhile or cost-effective [4]. The main limitations of the studies mentioned in a review by Menadue et al [4] were that NIV was applied only during exercise training, not during normal, day-to-day activities. Most devices lack portability and the ability to humidify during longer periods; these factors may also limit the use of NIV devices during PR. Additionally, the cost and time required to closely supervise exercise with such devices is prohibitive. The RACer-PAP, while still a prototype, offers a potential solution to overcoming these limitations and may provide patients with the ability to undertake activities of daily living in community settings due to its portability and humidification features. To our knowledge, this is unique in today’s assisted ventilation market. Prior to testing the device in a population of patients with COPD, assessing the device in healthy individuals was necessary.

Our study has focused on assessing the feasibility and utility of this new novel assistive ventilatory device in healthy individuals during exercise, with a view to extending this to a population of people with COPD. The prototype device has previously been investigated and found to be safe in several populations (including in healthy people at rest and in those with sleep apnea) [7,20], but has not previously been tested during exercise. During this study, we observed no adverse events with the RACer-PAP during either rest or exercise. There were no significant differences between pre- and posttest results for any cardiovascular, oxygenation, or exercise tolerance measures. There was a significant increase in the participants’ subjective assessment of their breathlessness during the 6MWT with the RACer-PAP in situ. While this difference was modest (a change in mean score of 2 to 3 on the Borg dyspnea scale), a change of 1 unit represents the minimal clinically important difference for this scale [21]. This increase in dyspnea score while using the RACer-PAP during the 6MWT in healthy individuals was anticipated by the research team prior to the study. The research team expected that healthy participants might find the increased inspiratory flow and expiratory pressure uncomfortable during exercise testing. An increase in the perceived work of breathing was also reflected in the subjective comments by participants. At rest, participants reported that the device was comfortable, although 5/15 participants (33%) reported that breathing at rest with the device was “harder.” During exercise, all participants (14/14, 100%) found it “harder” or “much harder” to breathe with the RACer-PAP, and 10 participants (10/14, 71%) rated their overall ability to exercise with the RACer-PAP “harder” or “much harder.” All participants’ dyspnea scores reverted to baseline within 2 minutes of ceasing the exercise test. All participants fully completed the exercise testing with the prototype RACer-PAP in situ and no participants requested the device be removed during the testing.

In people with COPD, it is possible that dyspnea and the perceived work of breathing may improve with the use of the RACer-PAP. One study of people with oxygen-dependent COPD found that using nasal high flow oxygen therapy (HFOT) increased tidal volume and end-expiratory lung volume and reduced respiratory rate at rest [22]. The mechanisms considered likely to be responsible for these changes were the probable reduction in anatomical dead space, the end-expiratory pressure of the HFOT device, and that the device functioned to match the participants’ increased flow demands to the flow provided by the device, reducing the airflow resistance and work of breathing. We hypothesize that the RACer-PAP has the potential to be equivalent to humidified high flow therapy and optimum end-expiratory pressures, potentially offering a viable option for improving outcomes in people with COPD.

Interestingly, in this study, there was a significant increase in FVC following exercise with the RACer-PAP in situ. This was not accompanied by an increase in FEV1 or FEV1/FVC ratio. Nonetheless, the actual mean difference in FVC with and without RACer-PAP in situ was only 160 ml (95% CI 19 ml-295 ml), which is unlikely to be clinically significant in healthy adults. It is possible that this data is either spurious or dependent on improvement in participant technique; it requires ongoing evaluation.

The use of other types of NIV during exercise in people with COPD has shown an unloading of both the inspiratory and expiratory respiratory muscle pumps [23], with the reduction in dyspnea being proportional to the respiratory muscle unloading [24]. Similarly, improvements in gas exchange and breathing pattern [24-27] have been demonstrated. Improved regional muscle perfusion [28] and decreased exercise-induced lactic acidosis [28] have been shown with the use of NIV during exercise training, resulting in an associated reduction in symptoms of muscle fatigue [28,29]. We hypothesize that the RACer-PAP may also provide similar benefits to people with COPD.

Dynamic hyperinflation (DH) of the lungs occurs in people with COPD during exercise when inspiration is initiated prior to complete exhalation of the previous breath, resulting in an increase in end-expiratory lung volume and subsequent restrictions on inspiratory capacity. Patients with airflow obstruction and subsequent gas trapping breathe at higher lung volumes, which requires a greater inspiratory effort to overcome elastic load. During exercise, an increase in respiratory rate, air trapping, expiratory flow limitation, and reduced expiratory time occurs. These changes can become significantly disabling and lead to exertional dyspnea. The use of strategies to reduce DH during exercise has been investigated, including pursed lip breathing, expiratory positive airway pressure devices, and NIV. A recent systematic review and meta-analysis [30] investigated the use of low-cost expiratory positive airway pressure (EPAP) devices, which increase resistance on expiration, increasing expiratory time and allowing for improved emptying of the lungs. While that study found that EPAP did not change DH, there was a reduction in respiratory rate. Limitations of the intervention included the use of face masks as the interface, a lack of additional inspiratory flow, no humidification, and that the EPAP levels were determined by the study authors, rather than by patient preference. It should be noted that if EPAP levels are too high, DH can increase dyspnea. Due to the low number of studies, low methodological quality, and small sample sizes of the studies in that review, further studies should be undertaken to assess the impact of EPAP on reducing DH and increasing exercise capacity in patients with COPD. The RACer-PAP offers an opportunity to further research in this area by offering a humidified, portable CPAP device with the addition of increased inspiratory flow to determine the impact on DH, exertional dyspnea, associated cardiovascular hemodynamics, and exercise capacity.

No limitations of the study design were identified by the research team. Several limitations related to the device were identified—participants’ comments about the prototype RACer-PAP highlight feasibility and utility issues. Prior to recruitment, participants were informed that the purpose of this initial study was to test the RACer-PAP during exercise in healthy individuals, with a view to determining the comfort and ease of use of the device prior to assessing the device in those with lung disease. Many participants commented on the device with this future objective in mind. It should be noted that none of the participants had previously used any form of positive pressure device and that their study experiences were not compared to any other NIV or positive pressure technologies. The participants made the following suggestions for future prototypes: reduce device weight and bulk, reduce length and size of tubing, improve device appearance (including the interfaces) to increase aesthetic discreetness when patients exercise or perform activities of daily living away from home, develop an alternative to the waist straps, increase the ease of self-administration of the RACer-PAP device, and develop an alternative to the current nasal interface.

Given the findings of this study, the research team hypothesizes that in those with COPD, the physiological effect of exercising with RACer-PAP in situ may reduce exercise-induced dyspnea, potentially leading to improvements in exercise and health-related QOL outcomes. We now propose to consider the application of the device in a small pilot feasibility study to assess the safety, feasibility, and utility of the device in a population of patients with moderate to severe COPD.

The current study has shown the prototype RACer-PAP to be safe and feasible to use during exercise in healthy participants. Further modifications to the device, as highlighted by the participants, are underway, and studies to assess the feasibility of use of the RACer-PAP with people with COPD have been proposed.

## Abbreviations

6MWT: 6-minute walk test
BP: blood pressure
COPD: chronic obstructive pulmonary disease
CPAP: continuous positive airways pressure
DH: dynamic hyperinflation
EPAP: expiratory positive airways pressure
ePARmed-X+: Electronic Physical Activity Readiness Medical Examination
FEV1: forced expiratory volume in 1 second
FVC: forced vital capacity
HFOT: high flow oxygen therapy
HR: heart rate
NIV: noninvasive ventilation
PAR-Q: Physical Activity Readiness Questionnaire
PEEP: positive end-expiratory pressure
PR: pulmonary rehabilitation
QOL: quality of life
RACer-PAP: rest-activity cycler-positive airways pressure
SpO_2_: peripheral oxygen saturation

## Data Availability

The data sets generated and analyzed during the current study are available from the corresponding author on reasonable request.
